# The relevance of formal and nonformal primary education in relation to health, well-being and environmental awareness: Bangladeshi pupils’ perspectives in the rural contexts

**DOI:** 10.1080/17482631.2018.1554022

**Published:** 2019-01-31

**Authors:** M. Mahruf C. Shohel, Andrew J. Howes

**Affiliations:** a School of Education, Aberystwyth University, Aberystwyth, UK; b School of Environment, Education and Development, The University of Manchester, Manchester, UK

**Keywords:** Relevance of education, environmental awareness, health and well-being, nonformal and formal education, school transition, rural contexts, Bangladesh

## Abstract

**Purpose:** This article reports part of a study focusing on young people’s transition from the nonformal to the formal education sector, and explores how the experiences of children and young people in remote formal and nonformal schools affect their awareness of issues of health, well-being and the environment. One of the main objectives of Bangladeshi extensive nonformal primary education, run by nongovernmental organizations (NGOs) in parallel with the formal system, is to prepare children outside schools to enter or re-enter the formal education sector. The study addresses the issue of educational relevance from pupils’ perspectives and looking at the implications for pupil transition between these two sectors.

**Method:** Interviews and observations of students and their classes were conducted in two contrasting rural high schools in different areas of Bangladesh, and their feeder primary schools.

**Results:** Where formal primary graduates focus more in high school on learning from their textbooks, nonformal primary graduates aim to put their knowledge into practice in their day-to-day life on a range of critical issues.

**Conclusion:** The results suggest an important contrast between nonformal and formal education sectors regarding students’ agency and knowledge of health and well-being, hygiene and environmental awareness in rural Bangladesh.

## Introduction

The importance of primary education in the overall education system of a country can hardly be overestimated. Historically and on a continuing basis, it is the community which normally establishes primary schools as social institutions to fulfil the educational needs of the next generation. The primary education in Bangladesh is free and predominantly government-run as universal primary education (UPE) is a legal commitment of the Government of Bangladesh. It works on a 5-year cycle for the 6–10-year age group. However, the government has not been able to deliver primary education to all school-age children, and even where schooling is available, many students have dropped out before completing their primary education cycle. Therefore, a parallel nonformal primary education (NFPE) system has developed in Bangladesh which provides children outside schools and dropouts with access to basic education (See Table 1 for detail below).

**Table I.** Situational differences between nonformal and formal primary schools.

Formal Primary Schools	Nonformal Primary Schools
Irregular supervision and less motivation	Regular supervision and strong motivation
Student: teacher ratio is high	Student: teacher ratio is low
Schools may be far away from home	Schools are close to home
Have rigid formal examination	Have flexible examination
Have physical punishment	Have no physical punishment
Have low level of extra-curricular activities	Have extra-curricular activities
Have no community participation	Have community participation
School hours are fixed	School hours are flexible
High cost of learning materials	Cost free education
Learners from different villages	Learners from neighbouring area
Extensive homework	Very little home work
Costly	Cost-effective
Follow national curriculum	Follow adapted national curriculum

Although the investment in education regarding the gross national product (GNP) is low compared to other South-Asian countries (Haq & Haq, 1998), primary education receives about half of the education sector budget (Rahman, Khan & Sabbih, 2016). By expanding enrolment and improving the quality of primary education, the Government expects to make a major contribution to a better-educated workforce in Bangladesh. Yet, problems remain. Of the 20 million primary school-aged children, four million are out of school, of whom at least half a million are dropouts (Shohel, 2010). As a result of reforms, enrolment levels are high, and gender equity has been reached but attendance and efficiency levels are average, and many disadvantaged children still do not attend school (BIDS, BBS and UNICEF Bangladesh, 2014).

It is in this context that nonformal education has developed, aiming to be suitable to the learning needs of the disadvantaged children. They require schools that are flexible in timing, are close to their home, and have a relevant curriculum, which will provide more than basic education. At the same time, schooling is a vital part of the development of basic skills for performing their roles and responsibilities in their families—skills that they can apply in real life situations as and when necessary (Shohel & Howes, 2007).

The nonformal primary school is one of the 12 types of schools that provide primary education in Bangladesh (Shohel, 2010). In the main, they are run by Non-Governmental Organizations (NGOs). Nonformal schools operate mainly in areas not served by either the government or private schools essentially to meet the educational needs of disadvantaged groups in the society. They usually follow an informal approach to suit the special needs of children from the marginalized groups (Centre for Policy Dialogue, 2001). One of the main objectives of nonformal primary education is to prepare students to enter or re-enter the formal education sector. However, there are other purposes too: nonformal primary education programmes aim to reduce illiteracy; contribute to the basic education of children, especially those from the poorest families and remote areas; promote the participation of girls in education; and support the Government’s universal primary education programme.

## Scope of the article

NGOs have their own models of nonformal education programmes. This article focuses on BRAC’s nonformal primary education model. BRAC is one of the largest non-governmental development organizations in the world in term of its numbers of employees and programmes it runs. It aims to alleviate poverty and empower the poor through micro-finance, health and education programmes in Bangladesh and other developing countries. These programmes are targeted to uplift the poor and female especially girls (BRAC, 2015).

This article does not address all aspects of formal and nonformal schools in the context of Bangladesh, but instead concentrates on health, well-being and environmental issues, addressing the following research question: How do the experiences of children and young people in formal and nonformal schools affect their awareness of issues of health, well-being and the environment?

## The impact of education on health

Education and health are arguably the most important aspects of developing human capital. Both make individuals more productive, and their economic value lies in the effects they have on productivity. Health and prosperity are positively correlated. Studies suggest that there are strong links between education and health (Feinstein, Hammond, Woods, Preston, & Bynner, 2003; Groot & van den Brink, 2004; Grossman & Kaestner, 1997; Wolfe & Zuvekas, 1997; Zimmerman, Woolf, & Haley, 2015). To some extent, the wealth of a nation is determined by the educational attainment and the health status of its population.

Education and health have the considerable impact on individual well-being (Groot & van den Brink, 2004). Studies suggest that “years of formal schooling completed is the most important correlate of good health” (Grossman & Kaestner, 1997, p. 73; Grossman, 1972). The positive association between education and health is well demonstrated through analysis of macro-level statistics: for example,

‘Education, health, nutrition and water and sanitation complement each other, with investments in any one contributing to better outcomes in the other’ (UN, 2003, p. 85).

Low-income countries have fewer resources to spend on publicly financed education and health care. Most individuals from low-income countries do not have the means to get the education and health care for themselves. In other words, investing in education and health provide the way out of the vicious cycle of poverty and are necessary to increase living standards.

Critical to our argument here is the significant impact that education has on health and well-being of young people. Children learn facts, concepts, manners, life skills, attitudes and competencies through education (Pring, 1995), and previous Bangladeshi studies explored how knowledge, values and attitudes are fostered through educational processes (Khan, 1995). Young people learn how to communicate, how to manage many aspects of everyday life, how to behave, how to use their knowledge and skills for generating incomes and how to take a role in a community setting. In this way, education is not only a medium of modifying behaviour or conditioning; it is a major means by which young people formulate morals and values. Education can help young people to understand the values and practices that contribute to healthy lives and communities—but of course that is not the only possible outcome. One of the most comprehensive and still definitive studies of these effects is by Wolfe and Zuvekas (1997) who identify five potential health and health-related effects of education:

1. A positive relation between one’s education and one’s health status;
2. A positive association between schooling and the health status of one’s family members (in particular on one’s children);
3. A definite link between one’s schooling and the schooling received by one’s children;
4. A positive contribution of education to the efficiency of choices (i.e., on smoking and on the use of health care);
5. A relation between schooling and one’s fertility on the probability of giving birth out of marriage as a teenager.

However, schooling does not guarantee these positive associations. In this article, we explore in greater depth the relationship between the experiences that children and young people have in school, and their awareness of significant issues relating to health, well-being and the environment.

## Methodology

This article is based on findings drawn from a research project focusing on the transition from the nonformal to the formal education sector in Bangladesh. Case study research was adopted, in order to develop a deeper understanding of the impact of schooling in the way that children and young people talk about and make sense of key aspects of the world in which they are growing up. The research was deliberately based on two very different sites in Bangladesh—Bogra and Norshingdi—chosen because of the different socio-economic background of the population. In addition, rivers split Bangladesh into different identities. To some extent, Bangladesh is a two-toned country between east and west side of Ganges (namely Padma and Megna rivers), and these sites are situated in different parts of the country.

The selection of sites in different parts of Bangladesh does not in itself increase the generalizability of results of this study (Gray, 2018). Our aim here is not to generate incontrovertible evidence, but rather a persuasive account based on close attention to young people’s realities and perspectives, using their own words as a basis for understanding their experience (Stake, 1995). However, by choosing sites in significantly different social locations in Bangladesh, the likelihood is greater that the issues they raise have relevance in many other similar contexts (Robson & McCartan, 2015).

Data were generated from rural parts of both districts using various research tools and techniques. A series of interviews with students and their teachers, and observations of teaching sessions were carried out in nonformal and formal primary schools, and in formal secondary schools. Six feeder primary schools (3 nonformal + 3 formal) from each geographical site were purposively selected. From each site one secondary school and one madrasa (a school with an emphasis on religion) were selected purposively based on the availability of enrolled nonformal graduates studying in Grade VI. Data used in this article derived from fieldwork conducted in two stages (December 2004–February 2005 and May–June 2005), with periods of analysis and writing in the UK.

## Methodological challenges

Presenting the challenges of carrying out the research provides a greater sense of the particular context of this research, and of the issues that are relevant to everyday life in these contexts. For example, gaining access to the field presented several different types of challenges. First, to get permission from BRAC was a big hurdle. It took 2 weeks to obtain official approval—and there was a continuing sense of suspicion and lack of trust from the NGO administration as well as formal school authorities. This process gives some indication of the system within which schooling operates, and in particular the sensitivities of officials to potentially negative findings from research in a context of competition for scarce resources.

In both research sites, access to transportation to reach the schools slowed down the fieldwork. There were physical challenges: travelling by public transport such as the bus to get to the study area with personal belongings and equipment was uncomfortable and exhausting. There was another delay waiting for the NGO workers to accompany the researcher to get to the selected schools. For that last stage of the journey, it was necessary to ride behind the worker on a motorbike, carrying a laptop, tape record and other essential stuff in a rucksack to prevent damage to the equipment. After returning from the field, the researcher experienced severe back pain almost every day—and again these experiences give a sense of the remoteness of the locations involved, and the challenges of everyday life for the participants in the research.

In addition, there were challenges based on preconceptions of the researcher’s position and relationship with the NGO as well as gender sensitivities within the socio-cultural context. As a male researcher, it was important to think carefully about how to work with female teachers and students, especially in one-to-one interviews in a culturally sensitive environment (Shohel, Jia, Jahan, & Roy, 2015). Suspicion of the researcher’s role by some of the respondents raises questions about the validity of the generated data, although these have been addressed through triangulation and through repeated visits to the field. There was another question of validity translating the transcripts of the interviews as they were carried out in Bangla, where proverbs and idioms were used embedded in the linguistic and cultural context of Bangladesh. Every language has its expression and elasticity which is sometimes very difficult to explain in another language.

These problems and difficulties are important to describe, not just in terms of the research process itself, but also insofar as they add to an understanding of the context in which the schools are located; geographically remote from the centre of the country, and woven into local hierarchies of organizational structure and administration, as well as socio-cultural gendered expectations and norms. It is in this context that the issue of schooling and attitudes to health and well-being were explored.

## Comparison between formal and nonformal primary schools

In order to present the data as precisely as possible, the following two sections offer a contrast regarding the learning environment, teaching and pupil perspectives between formal and nonformal primary schools. In the learning process, even small adjustments can bring about a considerable change in the overall learning environment, and it can be encouraging or deterring of children’s learning. Therefore, data for this article from the study has chosen environmental issues as investigation points for determining how the learning achievement of one system could be different from others and what the possible impacts of the classroom climate are on the student’s learning.

### Environment of formal primary schools

Classroom conditions always play an essential role in the teaching-learning process, especially with children. Psychologists suggest that young learners prefer to learn in a commotion-free, not too crowded, clean, open and benign environment (Corcoran, 2014; Shekh, 2005). In general, in Bangladesh, formal primary schools have a minimum of three classrooms and three teachers and an office-cum-teachers’ common room. Each classroom has benches for students, often with between five and 10 pupils on a bench. The average class size is 70 students and neither physical facilities, learning resources nor the teachers are capable of handling the numbers with good teaching practices (Japan Bank for International Cooperation, 2002). However, study shows that there is strong negative correlation between students learning outcomes and class size (Blatchford, Russell, Bassett, Brown, & Martin, 2007; World Bank, 2013)). Most formal primary school runs in two shifts. The first shift is for Grade I and II, and the other is for Grade III to V (Oxford Policy Management, 2006). Most of the primary schools can properly accommodate only about 60% of enrolled students (Chowdhury, Choudhury, & Ahmed, 2001). Students have to sit in crowded conditions in the classroom making it an uncongenial place for teaching and learning (Hossain, Imam, Amin, Rahman, & Ghosh, 2003). It was also found that classrooms are not always clean—papers, leaves and dust lie here and there on the floor (Observation). Evidence suggests (World Bank reports) that the Net Enrolment Rate (NER) has shot up in the last 15 years, which might also suggest that there is even more overcrowding. Usually, each formal primary school has a playground. According to Education Watch reports, over 90% of primary schools have a drinking water facility within its premises or in a nearby accessible place (Chowdhury, Choudhury, & Nath, 1999).

The following description from the field notes is typical description of one of the formal primary schools included in this study:

‘The school was a little away from the highway and had three classrooms and an office-cum-teachers’ common room. In the classroom where Grade V students sat, there were three windows and a door, which provided sufficient ventilation and light. There were twelve benches in three rows with enough space to accommodate around 40 students. Thirty-seven students were attending the class [It was found in the register books that 63 students were enrolled in Grade V]. There was some stuff on the floor here and there. There was no toilet facility in the school premises, and the learners had to go to the nearby bush to urinate. There was a small area in front of the school where the students played in their break times’ [Observation].

This description resonates with features that will be familiar to many educators with experience of schooling in remote areas of lower-income countries.

### Environment of nonformal primary schools

The nonformal primary school does not typically look like a formal high school regarding infrastructure and resources. Each BRAC nonformal school is a one-room school with a floor space of approximately 336 square feet. There are only 33 students and a teacher in a school. Two-thirds of the students are girls. The teacher, generally a female with at least 10 years of schooling, is chosen from the community where the school is situated. The same teacher teaches the same group of students from Grade I to V until they finish the nonformal primary education cycle. Though annual exam has been introduced in BRAC education system, continuous assessment of students’ performance is the main approach (BRAC, 1997). The classroom is very neat and clean, and students sit down on mats on the floor.

Again following field notes describe a typical school of this type:

‘This is a ‘one classroom, one teacher’ type of school. It was a rented tin-shade room with four windows and a door. Lighting and ventilation of the classroom were not so bad, but at the same time were not enough either [Previously this room was used as a living room and the area was smaller, but the owner rented it to BRAC and expanded it according to the school specification]. Students sat on mats. Thirty-three students were attending the class. No one was absent. They usually drink water from the landowner’s tube-well and use nearby urinal facilities. Four small houses surrounded this school. The school was just beside the main road. Easy communication was one of the reasons that were ensuring good supervision from BRAC and helped a lot to make this an effective nonformal primary school’ [Observation].

The following table summarizes some significant differences between the two types of primary schools observed in this research:

Differences between schools in the two fieldwork areas were much less marked, both in terms of these features, and also in terms of the data generated through interviews and observations. For this reason, data are drawn from both contexts largely without specifying the location, which also helps to risk issues of anonymity.

### Primary education and health behaviour

Before starting lessons in the nonformal primary (NFP) school, the teacher typically ensured overall cleanliness and arrangement of the classroom. During a social studies lesson, the teacher narrated a story about what happened to a person who ate some food that was left uncovered. The story was followed by the text on diarrhoeal disease and its causes and prevention. Thus, the teacher tried to speed the message of a particular lesson, using a story to drive it home [Observation]. In parents meetings too, ‘parents and teacher discuss the children’s progress, attendance, cleanliness and hygiene, the responsibility of parents towards their children, and any school problems requiring parental attention’ (BRAC, 1997, p. 3). The responsibilities taken by nonformal school teachers towards their pupils is remarked on in other research:

‘One of the most striking aspects of the schools is the role that the NFPE [nonformal primary education] teachers play in the student’s lives, both in and out of the classrooms. In the classroom, the teacher seems to adopt a role that is more facilitator than ‘teacher’ or ‘instructor’. She does not ‘instruct’- she discusses issues that range from social and moral values, personal hygiene, nutrition, etc. Outside the classroom she builds up a strong rapport with her students and their parents, visiting their homes, inquiring after their welfare, and motivating and advising them whenever needed’ (Begum, 1996).

The topics learnt in the nonformal primary school are related to everyday life such as cleanliness, habits, nutrition and health care. These, when taken home, can have a multiplier effect on the whole family. One of the respondents said,

‘‘I would go home and share something with my parents and siblings, and I encouraged my parents to send them [siblings] to school’’ [Interview].

Another respondent said,

‘‘I give more importance to wearing sandals when going to the toilet and cleaning my hands before eating. In health class, we were told to brush teeth before going to bed and keep away flies. I live with my grandmother and do some work at home, so all this is quite useful I think’’ [Interview].

Another said,

‘‘I learnt how to plant trees, wash my clothes regularly and keep my hair and nails clean. I also taught these things to my younger sister’’ [Interview].

Stronger parental involvement has been seen to have a positive impact on the efficacy of health messages (Wolfe & Zuvekas, 1997). Active parental involvement is another significant aspect of the nonformal school: the parental relationship with the school is much stronger. Whereas parents in the formal school are generally happy to leave the school to carry out the educational tasks; in the nonformal school, parents need to attend the parent-teachers meeting, and the school management committee (SMC), to discuss the students’ academic progress and any other issues. Often also, microcredit schemes are administered by the same NGO among the members of the same community. As a result, community mobilization and engagement establish a strong tie between the community and the nonformal primary school.

Regarding the primary curriculum, the basic difference between formal and nonformal schools is that formal curriculum is developed by NCTB (National Curriculum and Textbooks Board) at the national level. This formal national curriculum presents a standard form across the country, whereas the nonformal curriculum is an adapted version of the national curriculum which contains more “life skills” including awareness of health, nutrition, well-being and other social issues (Shohel & Howes, 2008).

The primary objective of lesson planning is that all activities and processes provide an educative environment for the learners (Orlich, et al. 1990). Therefore, the focus of the lesson plan is to set instructional objectives. Ordinarily instructional goals result in more effective teaching and testing and help teachers focus on what students should know at the end of the lesson. These objectives help teachers plan their teaching, organize instructions, stating expected behaviour, content, and outcomes, and provide clear direction for testing. During the fieldwork, it was found that only nonformal primary teachers plan lessons regularly. Teachers from formal primary schools said that their huge workload precludes them from doing so.

In interviews, it was found that young people themselves were very aware of the significance of schooling for their life course. It is worth noting the words of one of the respondents who attended a formal primary school but dropped out after 2 years of schooling. After re-enrolling at the nonformal school, she is now keen to continue her education. In her words:

“When I will finish Grade V, I would like to go to high school. I want to study more because it might help me to get a job. One day I would like to be a teacher” (Interview).

The pupil's experience of schooling in the nonformal school had shaped her intentions to go for further education, perhaps more so than her earlier experience in formal primary school. She can now imagine a future in which she herself is the teacher, and this pushes her towards high school as a next step.

### After transition to the formal high school

Students in formal secondary school who came from nonformal primary schools are acutely aware of health and hygiene issues. They were keen to share with the researcher what they had learnt from their Apa (teacher). Among their classmates, nonformal graduates are comparatively neat and clean though most of them wearing old clothes (Observation). Significantly, many of them express great unhappiness with their messy and damp high school environment especially the tube-well area and toilets. One of the female students reported that she never used the toilet in her present school because she heard from her mates that the toilets are very filthy and unhygienic and smelly. She is afraid that in using them, she might harm her health. The result is that every day she goes to school for nearly 7 hours without using the toilet, which can have dangerous consequences for health (UNICEF, 2005). In contrast, students who came from formal primary school hardly talk about their previous teachers in reference to their learning. And regarding their high school environment, most of them think that it is not too bad.

The classroom environment of both formal school and madrasa in Norshingdi were crowded to the point of being unhealthy. Observations on hot summer days took place in classrooms with no fans. Both teachers and students informed the researcher that there were cases of fainting due to the extreme temperature. When the researcher asked the head teacher why they do not place a fan in each room, he said that if they install fans in the classrooms, there is a possibility of theft. So he wants to tighten the security first at school building, and then to install fans in the classroom. The madrasa is also currently without fans—they explained they are going to extend the madrasa building soon and they will install fans after the expansion (Observation and Interview).

During observations, it was noted that the classroom was full of rubbish. Then in interviews with students, they were asked about it. They said they usually clean the classroom: the class-captain asks someone to do it on behalf of the whole class. However, that day class-captain did not ask anyone, and therefore, no one cleaned the classroom. One girl mentioned that they sweep it every alternate day. In comparison, nonformal students have had long experience of working in a clean environment (Observation). Most of the NGOs have health and family planning programmes in the community and continuous close supervision in their nonformal education programmes. They also have social awareness programme regarding good health and family planning. However, they have been working on those issues for a long time, using their expertise to provide training to their teachers about health and hygiene related issues.

Interestingly nonformal graduates in formal secondary school mentioned about the bigger space of the school; they like the big field attached to the school, the large school building, different classes and large student population. However, they also noted significant issues with basic hygiene the school environment, particularly relating to drinking water and toilets. One respondent told that,

‘‘The tube-well area is so filthy! Sometimes children leave loo near to the tube-well. They also use tube-well for cleaning them after shitting nearby which is disgusting and hazardous to public health because other people are using the same tube-well for drinking water” [Interview].

As she said,

“Even I can’t think how they do it” [Interview].

When a formal primary graduate was asked about the situation he said,

‘‘It is very normal. In my primary school, it’s even worse than our high school. We’re used to it’’ [Interview].

Interview data show that parents of the NFPE graduates who enrol in formal secondary schools have high expectations for their children in getting the higher education. However, because of the challenges associated with the transition, many of their children drop out before completing secondary school (Shohel, 2010). The data from interviews included here suggests that another part of the difficulty of transition is likely to relate to health issues originating in the school.

## Discussion

The results from the collected data suggest that there is a significant contrast between nonformal and formal education sectors regarding issues of health, hygiene and environmental awareness among primary graduates in rural Bangladesh. This contrast was made by focusing particularly on the issue of educational relevance from pupils’ perspectives, and then by looking for further evidence after transition into the formal secondary school.

Importantly, these differences were much greater than those identified between similar school types in the contrasting fieldwork locations. This feature of the study design strengthens the validity of the findings in respect of the type of schooling.

Nonformal primary education creates the second chance of education for the disadvantaged children, especially poor and girls to get access to basic education. Seventy per cent students of nonformal primary schools are girls, and in this way nonformal primary education addresses the issue of gender equity in education. In general, the nonformal schooling method pays much more attention to explanations of healthy and unhealthy behaviour, and there is a suggestion that these explanations are passed on to other members of the family (see also Shohel & Howes, 2008). From the point of view of educational relevance, the nonformal primary curriculum is more life-oriented then formal primary curriculum. The nonformal primary curriculum is based on the formal curriculum with particular emphasis on the needs of the target group.

Though the nonformal primary schools do not have permanent infrastructural facilities as they are rented and unlikely to continue as schools after completing nonformal primary cycle for a particular cohort, nevertheless well-trained teachers and close supervision from NGOs make its environment congenial (Shohel, 2012). This may well explain why when nonformal graduates move from that environment to formal secondary school with the less congenial atmosphere, they face a difficult or at least a troublesome transition.

Awareness about health and hygiene could contribute negatively during the transitional period. Data reveal that because of dirty toilets one female student did not used them since she started with that particular secondary school (Interview). It is obvious that physically she was harming her health. This lack of cleanliness contributes to a negative attitude to school, and that may lead the student to drop out from school (Shohel & Howes, 2005). Multiple cases found in different schools suggest that this is likely to be a continuing issue for the health and well-being of female children in particular in this context.

The benefits of the nonformal education intervention in the education system of Bangladesh and its citizens’ lives are both immediate and long-term. Nonformal education programmes provide literacy and life skills along with a social consciousness on the issues such as health care, hygiene, first aid, nutrition, sanitation, family planning, civic responsibilities, active citizenship etc. These have immediate effects on children’s “self-confidence” and on the capability to handle day-to-day affairs better and escape from exploitative social conditions and relations.

Interestingly, the awareness of environmental problems held by nonformal primary school graduates when they move to the formal high school may be a benefit to them, contributing to their life chances, to job prospects, to well-being, but perhaps not to educational achievement. The move from the tidy nonformal primary school to the messy formal secondary school creates the pupils’ awareness that this is wrong. The question then is at what point this awareness will become transformed into action, such that the young people concerned strengthen their identity as environmental activists (Shohel & Howes, 2011).

## Implications

With large non-participation and dropout in primary and secondary education and an overall low literacy level and poor health of the people, there remains a considerable need for nonformal education in Bangladesh. Generated empirical data reveals ways in which community-based nonformal primary education holds promise as an alternative means of providing basic education. In this way, nonformal primary education is complementary to formal primary education for the disadvantaged of the country and watering the seeds of inclusive primary education in Bangladesh to meet the targets of sustainable development goals (SDGs). Continuing exploration in this area will contribute to the emerging debate about nonformal education and its impact on shaping and reshaping future educational development of Bangladesh and beyond.

In the context of much higher net enrol rate (NER) in current years, whilst the participation rate has risen, nevertheless the issues that pertain in this data are likely to persist in a mass-schooling system unless consistent effort is made to position children and young people as having agency, and as needing to apply their knowledge with criticality, as we have seen these nonformal primary graduates doing in this article. Therefore, this research explores aspects of quality of education which most likely continue to have great significance, even in a country which is now doing much better in terms of the proportion of young people in school. The judgement on whether these findings continue to have validity and significance in this contemporary context can most readily be made by those who currently work and study within the system.

According to recent World Bank statistics, there are approximately 5 million young people currently not in school in Bangladesh. These statistics certainly stress the continuing relevance of nonformal education and of “Ananda Schools” located in poorest areas of Bangladesh to provide second chance education for reaching out-of-school children, which is a feature of policy in the last 15 years (World Bank, 2016). The question then is the extent to which this education carries the valuable characteristics of the nonformal education explored in this paper.

## Conclusion

This article has reviewed some of the literature on the link between educational experience and health behaviour, and drawn on data from a field study of schools in different parts of Bangladesh to extend these arguments, focusing mainly on the significant differences between formal and nonformal primary schools, which were much greater than the differences identified between schools in the contrasting fieldwork locations. The most significant differences between formal and nonformal schools in respect of their effect on attitudes to health and health behaviours are that nonformal primary graduates aim to put their knowledge into practice in their day-to-day life. In contrast, formal primary graduates appear to focus more on learning from their textbooks, and appear to lack the critical capacity to apply their knowledge in practice.

The findings are based on data from intensive fieldwork in a small number of locations in the two selected geographical locations, but there appears to be strong support for these findings in the related literature. Further study is needed to be carried out to see how knowledge about health, well-being and environment of primary graduates from both formal and nonformal sectors affect their perspectives and behaviours in the longer term.
